# LncRNAs: Architectural Scaffolds or More Potential Roles in Phase Separation

**DOI:** 10.3389/fgene.2021.626234

**Published:** 2021-03-31

**Authors:** Jie Luo, Lei Qu, Feiran Gao, Jun Lin, Jian Liu, Aifu Lin

**Affiliations:** ^1^Department of Obstetrics and Gynecology, Women’s Hospital, School of Medicine, Zhejiang University, Hangzhou, China; ^2^College of Life Sciences, Zhejiang University, Hangzhou, China; ^3^Department of Respiratory and Critical Care Medicine, The Second Affiliated Hospital, Zhejiang University School of Medicine, Zhejiang University, Hangzhou, China; ^4^Zhejiang University-University of Edinburgh Institute (ZJU-UoE Institute), Zhejiang University School of Medicine, Zhejiang University, Haining, China; ^5^Breast Center of The First Affiliated Hospital, School of Medicine, Zhejiang University, Hangzhou, China

**Keywords:** phase separation, lncRNAs, nuclear bodies, signaling transduction, therapeutics treatments

## Abstract

Biomolecules specifically aggregate in the cytoplasm and nucleus, driving liquid-liquid phase separation (LLPS) formation and diverse biological processes. Extensive studies have focused on revealing multiple functional membraneless organelles in both the nucleus and cytoplasm. Condensation compositions of LLPS, such as proteins and RNAs affecting the formation of phase separation, have been gradually unveiled. LncRNAs possessing abundant second structures usually promote phase separation formation by providing architectural scaffolds for diverse RNAs and proteins interaction in both the nucleus and cytoplasm. Beyond scaffolds, lncRNAs may possess more diverse functions, such as functioning as enhancer RNAs or buffers. In this review, we summarized current studies on the function of phase separation and its related lncRNAs, mainly in the nucleus. This review will facilitate our understanding of the formation and function of phase separation and the role of lncRNAs in these processes and related biological activities. A deeper understanding of the formation and maintaining of phase separation will be beneficial for disease diagnosis and treatment.

## Introduction

The assembly of liquid-liquid phase separation (LLPS) in cells mediates numerous membraneless compartments’ formation, such as stress granules (Wheeler et al., 2016; Wang M. et al., 2018; Gui et al., 2019), RNA-protein complexes, termed ribonucleoprotein (RNP) granules (Murakami et al., 2015; Pitchiaya et al., 2019), PGL-1/3 granules (Zhang et al., 2018), nuclear paraspeckles (Fox et al., 2018; Hupalowska et al., 2018; Yamazaki et al., 2018), and receptor clusters (Su et al., 2016). These compartments are involved in various physiological processes and pathological conditions. These two and three-dimensional membraneless organelles have well-defined boundaries, allowing specific biomolecules, such as proteins and nucleic acids, to be concentrated within liquid droplets and exchanged with the surrounding microenvironment (Banani et al., 2017). By creating distinct physical and unique biochemical compartments, phase separation facilitates temporal and spatial control of signaling transduction and biochemical reactions (Nott et al., 2015; Chong and Forman-Kay, 2016; Su et al., 2016). Phase separation transitioning from liquid to gel/solid implicates various central nervous diseases caused by aberrant aggregation of proteins common in amyotrophic lateral sclerosis (ALS) (Kim et al., 2013; Gasset-Rosa et al., 2019) and frontotemporal dementia (FTD) (Murakami et al., 2015). Dynamic liquid droplets formed by LLPS are believed to be driven by multivalent interactions between biomacromolecules containing intrinsically disordered regions (IDRs)/prion-like domains (PrLDs) or RGG/RG sequence (Kim et al., 2013; Banani et al., 2017; Chong et al., 2018). Those interactions always include charge-charge, pi-pi, and cation-pi interactions (Alberti et al., 2019). Those PrLDs and RGG sequences of RNA binding proteins (RBPs) possess small polar residues and aromatic, positively charged amino acids, which are critical elements for intermolecular interactions (Maharana et al., 2018; Alberti et al., 2019). Those RBPs contribute to the formation of RNP granules and nucleus paraspeckles through interaction with diverse RNAs in the manner of LLPS (Patel et al., 2015; Fox et al., 2018). In addition, LLPS is sensitive to its surrounding environment. Biophysical features of LLPS components (usually specific proteins and nucleic acids) and environmental factors (such as temperature, concentration of salt solution, pH, co-solute, the concentration of other macromolecules, and the modification of phase-separation-related components) have an enormous influence on intermolecular interactions between RBPs and RNAs (Brangwynne et al., 2015; Nott et al., 2015; Reichheld et al., 2017; Franzmann and Alberti, 2019). Post-translational modifications (PTMs), such as phosphorylation (Larson et al., 2017; Zhang et al., 2018), methylation (Qamar et al., 2018; Ryan et al., 2018), ubiquitination (Dao et al., 2018), and SUMOylation (Jin, 2019; Qu et al., 2020) of proteins and m6A modification of RNA (Ries et al., 2019), modulate LLPS formation through regulating protein-protein or protein-RNA interaction, which are affected by the net charge distribution of those molecules.

As a member of phase separation, RNA cooperates with protein partners to drive LLPS formation and modulates the properties of droplets (Huo et al., 2020). Emerging pieces of evidence have reported that RNA not only serves as a scaffold in phase separation due to their abundant secondary structures (Jain and Vale, 2017; Fay and Anderson, 2018; Maharana et al., 2018), but also for their ability to decrease the viscosity of protein components and promote the diffusion of protein components (Elbaum-Garfinkle et al., 2015). Long non-coding RNAs (lncRNAs) are longer than 200 nt in length and unable to code proteins, but they play critical roles in cell metabolism and tumor development, largely depending on their subcellular localization (Zhang et al., 2014). Nuclear lncRNAs regulate transcription, epigenetic modification, and splicing processes of mRNAs (Schmitt and Chang, 2016; Tang et al., 2017). Evidence reveals that lncRNAs regulate mRNA translation and degradation by complementary base pairing and serve as an RNA sponge by interacting with the miRNA in cytosol (Yoon et al., 2013). Our previous studies have revealed that lncRNAs coordinate diverse signal transduction pathways, such as PIP3, HIF1-α, Hippo, Hedgehog, and NF-κB, to promote tumor development (Lin et al., 2016, 2017; Zheng et al., 2017; Sang et al., 2018). Compared to small RNAs, lncRNAs are more capable of providing binding sites for RBPs involved in phase separation (Chujo et al., 2016; Chujo and Hirose, 2017; Fox et al., 2018; Yamazaki et al., 2018). The classical paraspeckles, which are mainly constituted by lncRNA *NEAT1* and numerous RBPs, sequester component proteins and RNAs in the nucleus to mediate gene expression by extensive polymerization and multivalent interaction of LLPS components (Fox et al., 2018; Yamazaki et al., 2018). However, further investigation is needed to understand how lncRNAs coordinate phase separation in different subcellular localization to contribute to diseases (such as degenerative diseases) and tumor development.

**FIGURE 1:**
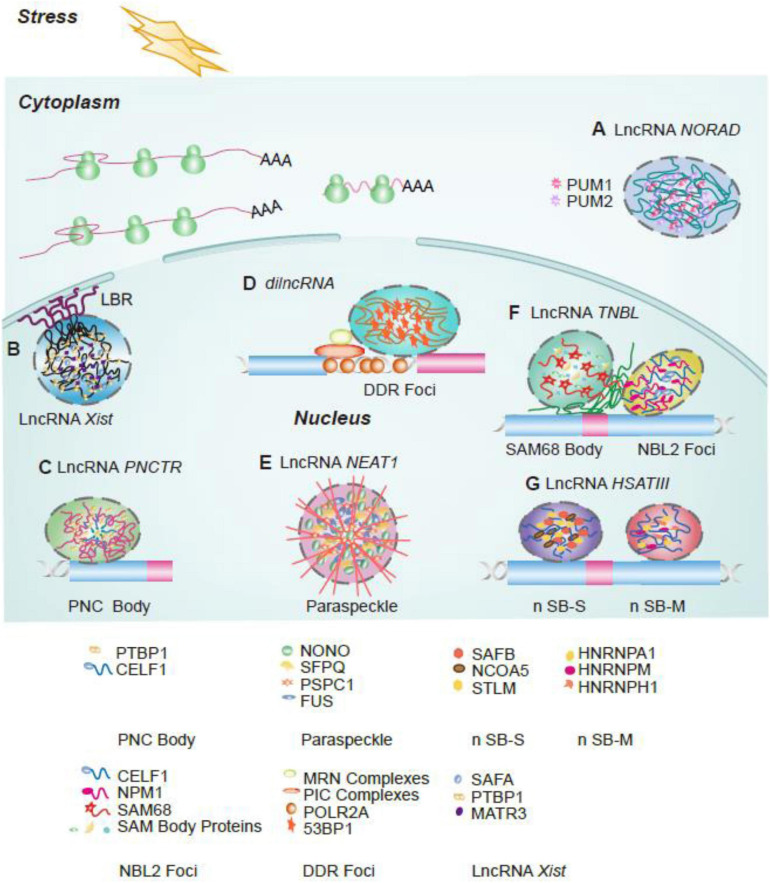
The graphical abstract of phase-separation related LncRNAs involved in cellular function. **(A)** LncRNA *NORAD* functions as a multivalent binding platform for PUM1/2 proteins in cytoplasm; **(B)** LncRNA *Xist* mediates X chromosome silence and subsequently drives interaction between inactivated X chromosome and Lamin-B receptor (LBR); **(C)** LncRNA *PNCTR* sequesters PTBP1 in the perinucleolar compartment (PNC) and modulates splicing regulation function of PTBP1 protein; **(D)** *DilncRNA* synthesized at DSB foci and coordinates DDR proteins to promote the formation of DDR foci to response to DBS; **(E)** LncRNA *NEAT1* functions as scaffolds to recruit CARM1, PSPC1, and p54nrb proteins to regulate cell differentiation and embryo development in paraspeckle; **(F)** LncRNA *TNBL* is accumulated as a perinucleolar aggregate at NBL2 loci and close to SAM68 body and is involved in genome organization, splicing regulation, and mRNA stability, respectively; **(G)** LncRNA *HSATIII* is involved in two nuclear bodies, n SB-M and n SB-S, formation to respond to thermal stress.

This review summarized current advances about phase separation and related lncRNAs in nucleus and cytosol during numerous biological processes (Figure 1). We have also summarized the lncRNAs referred to in this review (Table 1). Finally, potential therapeutic targets in phase-separation-related lncRNAs and phase separation components during disease development are also summarized.

**TABLE 1:** The LncRNAs referenced in this review.

**LncRNAs**	**Subcellular localization**	**Biological function**	**References**
LncRNA *LINKA*	Cytoplasm	Hyperactivate AKT, HIF1-α signaling pathway, and downregulate antigen presentation related genes to promote drug resistance and immune escaping and remodel glycolysis reprogram of cancer cells.	Lin et al., 2016, 2017; Hu et al., 2019
LncRNA *BRCA4*	Nuclear	Coordinate hippo and hedgehog signaling pathways to aberrantly regulate glycolysis and advance breast cancer development.	Xing et al., 2014; Zheng et al., 2017
LncRNA *CamK-A*	Cytoplasm	Assist the Ca2^+^ signaling pathway to aberrantly regulate glycolysis and remodel tumor microenvironment.	Sang et al., 2018
LncRNA *NEAT1*	Nuclear	Function as a scaffold for paraspeckle components and sequester specific proteins (such as CARM1) promotes cell differentiation and embryo development. Attenuate activation of p-53 and confer cancer cell drug resistance (LLPS).	Chen and Carmichael, 2009; Adriaens et al., 2016; Fox et al., 2018; Hupalowska et al., 2018; Yamazaki et al., 2018
LncRNA *MAYA*	Cytoplasm	Mediate heterodimerization of ROR1 and HER3 and promote activation of YAP, thus facilitating breast cancer bone metastasis.	Li et al., 2017
LncRNA *HOTAIR*	Nuclear	Assist PRC2 complex to recruit to histone and be responsible for the silence transcription of HOXD gene.	Rinn et al., 2007
*LincRNA*	Nuclear	Bind to a series of chromatin-modifying proteins to maintain the pluripotent state of ESCs.	Guttman et al., 2011
LncRNA *NORAD*	Both nuclear and cytoplasm	Assemble a topoisomerase complex at targeted chromatin foci to stabilize genome (Nuclear). Function as a multivalent binding platform for PUM1/2 proteins, and thus maintaining genomic stability (LLPS).	Lee et al., 2016; Tichon et al., 2016; Munschauer et al., 2018
LncRNA *Xist*	Nuclear	Recruit epigenetic factors to chromosome loci and mediate X chromosome silence (LLPS).	Heard and Disteche, 2006; Moindrot et al., 2015; Cerase et al., 2019
LncRNA *TCF7*	Nuclear	Recruit epigenetic factors SWI/SNF promoting TCF expression, thus activating Wnt pathway to promote self-renewal of liver cancer stem cells.	Wang et al., 2015
eRNA	Nuclear	Bind to multiple TFs and coactivator to alter the chromosomal architecture and thus regulating gene expression.	Li et al., 2013; Liu et al., 2014; Pnueli et al., 2015
LncRNA *GATA6-AS*	Nuclear	Recruit and inactive epigenetic factor LOXL2 and regulate endothelial gene expression and angiogenic activity in responding to hypoxia.	Neumann et al., 2018
LncRNA *AGPG*	Both nuclear and cytoplasm	Stabilize PFKFB3 by blocking its ubiquitination and degradation thus promoting glycolysis in cancer cells.	Liu et al., 2020
lncRNA *HOXB-AS3*	Both nuclear and cytoplasm (according to genecard)	Encode peptide HOXB-AS3 regulating splicing of pyruvate kinase M (PKM) and thus reprogramming glucose metabolism.	Huang et al., 2017
*LOC100507537/LINC00948*	Sarcoplasmic reticulum membrane (according to genecard)	Encode peptide activating the SERCA pump to promote Ca2^+^ up-taking into sarcoplasmic reticulum (SR) and enhancing muscle contractility.	Anderson et al., 2015; Nelson et al., 2016
LncRNA *meiRNA*	Nuclear	Function as an architectural scaffold promoting the formation of sme2 chromosomal loci (phase droplet) and mediate pairing of homologous chromosomes (LLPS).	Shichino et al., 2014; Ding et al., 2019
LncRNA *HSATIII*	Nuclear	Function as an architectural scaffold interacting with two hnRNPs to promote nuclear stress bodies formation upon thermal stress exposure (LLPS).	Aly et al., 2019
LncRNA *PNCTR*	Nuclear	Function as an architectural scaffold sequestering PTBP1 in the perinucleolar compartment, thus modulating splicing of PTBP1 protein and promoting cancer cell survival (LLPS).	Yap et al., 2018
LncRNA *TNBL*	Nuclear	Accumulate as a perinucleolar aggregate at NBL2 loci and close to SAM68 body and thus responding to nuclear functions and RNA metabolism (LLPS).	Dumbovic et al., 2018
*DilncRNA*	Nuclear	Is synthesized at DSB foci and coordinates DDR proteins to promote the formation of DDR foci to respond to DBS (LLPS).	Pessina et al., 2019
LncRNA *MajSAT*	Nuclear	Functions as a scaffold promoting HP1α and SAFB to form PCH foci in Pericentromeric heterochromatin (LLPS).	Huo et al., 2020
LncRNA *TERRA*	Nuclear	Considering the enrichment of LncRNA *TERRA* in APB and interaction between LncRNA *TERRA* and epigenetic modification factors and RBPs, LncRNA *TERRA* may also play a functional role in the telomere foci by providing a platform for multiple proteins interaction (LLPS).	Min et al., 2019

## Phase Separation

The membrane organelles in eukaryotic cells are well-defined by their membrane-boundaries which provide relatively independent compartments for their specific function (Hyman et al., 2014; Nott et al., 2015; Alberti et al., 2019). For example, endoplasmic reticulum (ER) is involved in the processing of protein and the synthesis of lipids; Golgi apparatus participates in the processing, sorting, and transporting of proteins. Lysosomes function as the cleaning machines for misfolding and pathological proteins; mitochondria provide cellular fuel. However, how do membraneless organelles assemble proteins, nucleic acids, and other molecular components into phase separation? What are the roles of these membraneless organelles in biomolecules metabolic processes, stress sensing, signaling pathways transduction, and gene expression regulation remain largely unknown. Since Hyman and Brangwynne first reported the formation of germline P granules by phase separation in worm embryo cells in 2009 (Brangwynne et al., 2009), the number of studies on phase separation touching myriad cellular functions have increased significantly.

The regulation of gene expression is a prominent event in healthy and diseased states and involves many factors (such as enhancers and coactivators). Recent studies suggested that gene regulation is always accompanied by phase separation assembled by numerous IDR proteins (Guo et al., 2019). Using live-cell super-resolution light-sheet imaging, a previous study found that mediator coactivator coordinates RNA polymerase II (RNA pol II) to regulate the assembly of mediator cluster at enhancer, thus activating gene expression (Cho et al., 2018). Typically, enhancers can activate promoters within the locus (Palstra et al., 2003). Those phase separation-mediated enhancers cause gene bursting expression. Transcriptional factors (TFs) MED1 and BRD4 condensate at super enhancers’ (SEs) foci to coactivate gene transcription. This phase separation formed by SEs and TFs confers robust gene expression, which could explain why cancer cells acquire large SEs at driver oncogenes and results in bursting gene expression from a new perspective (Sabari et al., 2018). The composition of amino acids of TFs’ activation domain in mammalian OCT4 and yeast GCN4 is vital for forming phase separation. Phase separation also coordinates multiple signaling pathways (such as estrogen receptor (ER) and Yes-associated protein (YAP) signaling axis) to respond to stress (Boija et al., 2018; Cai et al., 2019). Changes in the components of phase separation often have an impact on their function. Phase separation formed by the histidine-rich domain (HRD) of cyclin T1 and DYRK1A contributes a lot to phosphorylated C-terminal domain (CTD). Disruption of HRD interaction downregulated gene expressions (Lu et al., 2018). Phase separation accumulated at chromatin foci is significantly dependent on the conformation of nucleosomes. A loose conformation of nucleosomes means the activation of chromosomes, while tight condensation suggests the formation of heterochromatin. Heterochromatin protein 1 (HP1) is known to finely tune heterochromosome phase separation by participating in weak multivalent interaction of nucleosomes (Larson et al., 2017; Sanulli et al., 2019). H1 histone and the 10n + 5 inter-nucleosome spacing promotes the phase separation of chromatin and decreases dynamics in droplets (Gibson et al., 2019). Those models of heterochromatin formation provide a new perspective to understand phase separation in regulating the conformation of chromatin. Regulation of gene expression by phase separation broadens our understanding of the mechanism of aberrant expression at the transcriptional level in numerous diseases, facilitating the development of new strategies to identify key components involving the formation and maintenance of phase transition. A novel CRISPR-Cas9-based optogenetic technology was used to explore the formation of droplets impacted by the chromatin microenvironment. This study suggested that phase separation is preferentially formed at low-density genomic regions and promotes genomic rearrangements, thus contributing to the activation of gene expression. On the contrary, at high-density genomic regions, small droplets ultimately dissolve, contributing to the disappearance of phase separation (Shin et al., 2018). These pieces of evidence indicated that the structure of genome and phase separation affected each other, both of which have an enormous impact on gene expression. The existence of phase separation could explain the aberrant patterns of gene expression well.

Phase separation transition from a liquid to a gel or solid leads to degenerative neurological diseases (Wang and Zhang, 2019). Heterogeneous nuclear ribonucleoproteins (hnRNPs) containing IDRs or PrLDs, such as FUS, hnRNPA1, or TAR DNA-binding protein 43 (TDP-43), are found rich in many aging-associated diseases (Kim et al., 2013; Patel et al., 2015; Gui et al., 2019; Mann et al., 2019). Tau droplets formed by phosphorylated or mutant Tau with IDRs undergoing LLPS contributes to Alzheimer’s disease (Wegmann et al., 2018). Fused in sarcoma (FUS) is an RNA-binding protein involved in RNA transcription, splicing, transporting, and translation. With classical IDR and low-complexity domain (LCD), FUS protein transitions from a liquid to aggregated state, promoting LLPS formation at the sites of DNA damage, which is associated with ALS (Patel et al., 2015). As membraneless organelles, phase separation can sequester specific components to accelerate or inhibit unique cellular function, and thus advance disease development. Mislocalization and aberrant aggregation of misfolded TDP-43 sequester importin-α and Nup62 in the cytoplasm. Depletion of importin-α and Nup62 in the nucleus induces RnaGap1, Ran, and Nup107 mislocalization, thus promoting cell death and causing advanced ALS and FTD (Gasset-Rosa et al., 2019). Phase separation can also contribute to the development of degenerative neurological diseases. Degenerative neurological-disease-related mutations can also affect the formation of phase separation. Recent studies reported that ALS/FTD related mutation-induced FUS phase transition from liquid droplets to irreversible hydrogels, which impairs RNP function and advances disease (Murakami et al., 2015; Patel et al., 2015). Similarly, the ALS-related mutations in the TDP-34 C-terminal domain (CTD) disrupt phase separation and impair interaction within the phase droplets, which promotes LLPS transition into solid aggregation, thus aggravating the ALS condition (Conicella et al., 2016). Mutations in prion-like domains in hnRNPA2B1 and hnRNPA1 also contribute to ALS (Kim et al., 2013). Elucidation of the exact mechanism involved in the molecular properties, formation, regulation, and function of membraneless organelles can help us explore novel therapeutic approaches to treating aging-related disorders. Optogenetic approaches used in controlling phase separation formation of TDP-43 reveals that LCD of TDP-43 are competitively bound by RNA. And oligonucleotides composed of the TDP-43 target sequence can moderate the neurotoxicity caused by aggregation of TDP-43 (Mann et al., 2019). Dysregulation of phase separation in aging-related protein accelerates the malignant transition, but is not a one-way process. Extensive exploration of those processes helps us better understand the development of aging-related diseases.

## LncRNAs in Cell Behavior

Nearly 98% of human genome encodes as non-coding RNAs (ENCODE Project Consortium et al., 2007; Schmitt and Chang, 2016). For such a large amount of non-coding RNAs (ncRNAs), their cellular function has intrigued many researchers. According to the size, ncRNAs are divided into small ncRNAs and long ncRNAs (Brosnan and Voinnet, 2009; Liu et al., 2019). LncRNAs are poorly conserved in terms of their nucleotide sequences, even though they can be found in many species (Johnsson et al., 2014; Beermann et al., 2016). Secondary structures of lncRNAs enable them to interact with DNAs, proteins, and RNAs, allowing them to participate in multiple cellular processes (Fernandes et al., 2019).

Emerging evidence has revealed that different subcellular localizations of lncRNAs engage in numerous biological processes, including regulation of gene transcription, chromatin remodeling, cancer-related signaling pathways, and organism development (Li et al., 2017; Zheng et al., 2017; Sun et al., 2018; Sarropoulos et al., 2019). Nuclear-localized lncRNAs are involved in transcriptional and post-transcriptional modification and chromatin organization (Sun et al., 2018). The intended transcriptional regulation function of lncRNAs largely relies on multiple interactions between lncRNAs and other molecules (such as DNA, proteins, and RNAs). LncRNA *HOTAIR* assists the PRC2 complex to accumulate at histone and is responsible for the silence of HOXD gene (Rinn et al., 2007). Researchers identified dozens of lncRNAs involved in binding to a series of chromatin-modifying proteins to maintain the pluripotent state of stem cells (Guttman et al., 2011). LncRNA *NORAD* assembles a topoisomerase complex at targeted chromatin foci to stabilize the genome (Munschauer et al., 2018). Classical lncRNA *Xist* mediates X-chromosome inactivation by recruiting protein complexes to repress epigenetic marks and encompass the X-chromosome (Heard and Disteche, 2006). LncRNA *TCF7* recruits SWI/SNF5 complexes to TCF7 promoter to mobilize nucleosomes and remodel chromatin conformation, promoting liver cancer stem cells self-renewal (Wang et al., 2015). All studies mentioned above suggested that lncRNAs are vital for gene expression at the epigenetics level. LncRNAs also act as local regulators to influence the expression of nearby genes by *cis* regulation (Guil and Esteller, 2012; Engreitz et al., 2016). Enhancer RNAs (eRNAs) transcribed from bidirectional ncRNA can bind to multiple TFs and coactivators to alter the chromosomal architecture and regulate gene expression (Li et al., 2013; Liu et al., 2014; Pnueli et al., 2015). The emerging roles of eRNAs significantly extend our understanding of the function of gene transcription regulated by lncRNAs.

Dysregulation of lncRNAs in cells and tissues is associated with malignant transformation and various pathological processes (Xing et al., 2014; Neumann et al., 2018), which is always coordinated by multiple classical signaling pathways. Our previous studies have suggested that lncRNA *LINK-A* is involved in breast cancer drug resistance (Lin et al., 2017), hypoxia (Lin et al., 2016), and immunosuppressive microenvironment (Hu et al., 2019). LncRNAs *CamK-A*, *BRCA4*, and *AGPG* wires up NF-kB (Engreitz et al., 2016), Hippo and Hedgehog (Zheng et al., 2017), and PFKFB3 glycolytic enzyme complexes (Liu et al., 2020), respectively, to remodel glucose metabolism and tumor microenvironment, promoting tumor development. LncRNAs are characterized to have specific tissue distribution, which implies their functional role in development and differentiation. Through genome-wide analysis, Luo et al. (2016) found that divergent lncRNAs regulate about 168 genes coding transcription factors and developmental regulators in embryonic stem cells (ESCs), which implies lncRNAs may be developmentally regulated (Schmitz et al., 2016). The developmental lncRNAs atlas constructed by Sarropoulos et al. (2019) revealed that lncRNAs show species specificity and dynamic expression pattern from early organogenesis to adulthood suggesting that the time, lineage-, and organ-specific lncRNAs are responsible for specific functions during organogenesis and organism development. The functions of lncRNAs are much more than what has been mentioned above. Recent advances in deep-sequencing technologies have identified that some lncRNAs have the ability to encode functional peptides (Anderson et al., 2015; Nelson et al., 2016; Huang et al., 2017). And more interestingly, coordination of the phase separation formation by lncRNAs has been reported in many recent studies.

## LncRNAs and Phase Separation

The essential features of phase separation are mainly determined by their components. Phase separation related to the regulation of gene expression always takes place in the nucleus. Nuclear bodies, clustering factors, super-enhancers, and chromatin foci are often related to phase separation and transcriptional regulation. Those phase separation membraneless organelles are storage compartments for many RNAs and RNA binding proteins. RNAs involved in the formation of phase separation function as scaffolds and eRNA, whereas phase separation, in turn, impacts the behavior of RNAs, such as synthesis (Pefanis et al., 2015; Nair et al., 2019). The nucleoplasm is a natural pool abundant with diverse membraneless nuclear bodies regulating gene expression (Chujo and Hirose, 2017; Ninomiya and Hirose, 2020), especially paraspeckle and chromosome loci. Those nuclear bodies are usually composed of multiple RNAs and RBPs containing PrLD and RGG sequence (Van Treeck and Parker, 2018; Alberti et al., 2019). In phase separation nuclear bodies, RNAs critically regulate the phase behavior of RBPs with PrLD. Different RNA/protein ratios exert different influences on phase separation transition. To some extent, RNAs act as a buffer in the nucleus where high RNA concentrations keep RBPs soluble (Van Treeck and Parker, 2018). Changes at RNA levels or RNA binding abilities of RBPs cause aberrant phase transitions (Maharana et al., 2018). This makes us consider that RNAs in phase separation can competitively bind with proteins containing IDR, which attenuates protein self-aggregation. This implies that RNAs with a bigger size, especially lncRNAs, may be more efficient in buffering phase separation.

In addition to the buffering function in the nucleus, many lncRNAs often serve as scaffolds for nuclear bodies’ formation. LncRNAs act as seeds to recruit specific component proteins by RNA-proteins interactions (Fox et al., 2018). Those RBPs always recruit additional proteins to induce the formation of LLPS and control gene expression under certain stimulations. Among those nuclear bodies, paraspeckle is a sound model system with well-defined RNAs and protein components for the study of phase separation (Fox et al., 2018). LncRNA *NEAT1* has good architectural functions to provide a scaffold for multiple RNA-binding proteins (RBP) in paraspeckles construction (Adriaens et al., 2016). Gene expression is affected by the size and number of paraspeckles, which can sequester specific RBPs and/or RNA away from nucleoplasm to achieve the regulation (Chen and Carmichael, 2009). The paraspeckles formation is similar to cytoplasmic stress granules, which are another membraneless organelle (Fox et al., 2018). Both paraspeckles and stress granules can respond to cellular stress and function by sequestering specific components to regulate stress response-related gene expression. Those membraneless organelles seem to be more flexible than compartmentalized organelles during stress response due to their dynamic disassembling and assembling. The aberrant gene expression in paraspeckle is often associated with cancer progression (Adriaens et al., 2016). Another typical transcriptional element enhancer is also responsible for gene bursting transcription. Phase separation model suggested that super-enhancers (SEs) consisted of cluster enhancers involved in high transcriptional activity of related genes (Pefanis et al., 2015; Hnisz et al., 2017). In the SEs foci, phase separation regulates the degradation and accumulation of eRNAs, which finally affects the stability of the genome (Pefanis et al., 2015). The function of eRNAs and SEs in phase separation provides us new insights into the regulation of gene expression. In addition to the regulation of nuclear body formation by lncRNAs, delineating the phase behavior mediated by lncRNAs beyond the nucleus can shed light on the impact of cytoplasm condensations on signaling transduction and cellular metabolism. LncRNA *NORAD* retains PUM1/PUM2 protein in the cytoplasm to form RNP granule, leading to chromatin instability in response to DNA damage. In this study, LncRNA *NORAD* functions as a platform to sequester PUM1/PUM2, negatively regulating PUMILIO activity in the cytoplasm. This leads to elevated key mitotic, DNA repair, and recruitment of DNA replication factors. In this RNP granule, lncRNA *NORAD* may coordinate interferon response pathway proteins IFIT1/2/3/5 to regulate this process (Lee et al., 2016; Tichon et al., 2016). Although little is known about the role of lncRNAs in cytoplasm phase separation, one can speculate multiple potential functions of lncRNAs in forming and maintaining phase separation and many other biological processes.

### LncRNAs Modulate Phase Separation in Nucleus

In mammalian cells, there are various nuclear bodies. They are mainly involved in the regulation of gene expression by transcriptional epigenetic modification. Chromosome homologous pairing and separation, chromatin remodeling, and RNA splicing are common events in the nucleus, often mediated by phase separation. Many nuclear bodies are well-defined by the enrichment of specific proteins and RNAs (Ninomiya and Hirose, 2020). Beyond function as architectural RNAs, lncRNAs also serve as eRNAs that exist in phase separation droplets (Chujo et al., 2016). The mechanism of how those nuclear bodies exert their functions remains poorly understood. There may be three reasons. First, those nuclear bodies function as a reaction tank sequestering specified molecules, such as enzymes and their substrates. Second, they act as a sequestering compartment, which can condensate specific molecules and protect them from degradation, or sequester from nucleoplasm, to impair their function. Third, they can form an organizational hub that anchors chromatin loci to remodel chromatin and regulate gene expression.

#### Paraspeckle

Paraspeckle was first reported in 2002 as a marker of paraspeckle component proteins 1(PSPC1) and subsequently found to be mainly localized in mammal cell nuclei (Fox et al., 2002). In addition to PSPC1, paraspeckle consists of over 40 different proteins and the structure lncRNAs *NEAT1* (Mao et al., 2011; Fox et al., 2018). *NEAT1* depletion completely abolished the formation of paraspeckle (Sasaki et al., 2009; Shevtsov and Dundr, 2011). PSPC1 was first reported to be enriched in paraspeckle, but later it was reported that PSPC1 together with NONO and SFPQ were dispensable for paraspeckle formation (Sasaki et al., 2009; Naganuma et al., 2012). EM and super-resolution microscopy have revealed that paraspeckle is a spherical shape with a shell and core. 3′ and 5′ ends of lncRNA *NEAT1* are extended out of paraspeckle in the form of bundles (West et al., 2016). Once formed, paraspeckle sequestered specific RNAs and proteins to alter the levels of those components, changing the cellular processes (Prasanth et al., 2005; Chen and Carmichael, 2009). Paraspeckle in the nucleus participates in many cellular processes, usually related to stress response and cancer. P53 regulates the transcription of *NEAT1*, which promotes the formation of paraspeckle and confers breast cancer cell drug resistance (Adriaens et al., 2016). High level PSPC1 expression in cancer cells activates the TGF-β pathway and promotes metastasis (Salvador and Gomis, 2018). Recent studies link paraspeckle to mitochondrial homeostasis against the stress response. Classical paraspeckle-mitochondria crosstalk provides a nice model for understanding the role of *NEAT1* and paraspeckle in cancer and neurodegeneration (Nishimoto et al., 2013; Adriaens et al., 2016; Fox, 2018; Wang Y. et al., 2018).

As a classical nuclear body, paraspeckles are involved in gene expression regulation and retention of mRNAs and proteins (Hirose et al., 2014; Wang Y. et al., 2018). Recent studies wired paraspeckles with phase separation and found paraspeckle are more likely to form droplets (Fox et al., 2018). Previous studies revealed that many paraspeckle proteins (such as RBM14 and FUS) containing IDR are responsible for phase separation formation (Hennig et al., 2015; Patel et al., 2015; West et al., 2016; Shin et al., 2017). During preimplantation development of mouse embryo, activation of histone by the histone coactivator associated arginine methyltransferase 1 (CARM1) is necessary for the upregulated expression of a subset of pluripotency genes. The function of CARM1 is maintained by paraspeckle integrity and dependent on lncRNA *NEAT1* and NONO (Hupalowska et al., 2018). A specific sequence in 3′-UTR of RNA makes it prone to be bound with paraspeckle components. The latest study reported that paraspeckle lncRNA *NEAT1* and four major proteins are responsible for retaining circadian mRNA to regulate gene expression at post-transcriptional level (Torres et al., 2016). The size and number of paraspeckles significantly affect gene expression. In contrast, the assembly of paraspeckle is mainly determined by the level of *NEAT1* and components proteins such as SFPQ and FUS. An earlier study revealed that the bigger a paraspeckle becomes, the more SFPQ is needed. The decreasing level of SFPQ in the nucleus altered the targeted gene expression, which also occurred in other nuclear bodies (Chen and Carmichael, 2009; Imamura et al., 2014; Wu et al., 2016). As the paraspeckle component proteins, such as SFPQ and NONO, are involved in pri-miRNA processing, sequestering both of those proteins can affect miRNA processing (Jiang et al., 2017). Studies indicate that expression and mutation of core paraspeckle structure *NEAT1-2* are often related to multiple cancers (Fujimoto et al., 2016; Rheinbay et al., 2017). All of this evidence indicates that phase separation in paraspeckle can regulate gene expression and RNA-related processes, promoting disease and cancer development.

Paraspeckles are involved in various cellular processes. The core structure of lncRNA *NEAT1* is responsible for building paraspeckles. The protein components of paraspeckles usually contain IDR, which promotes the formation of phase separation. Therefore, investigating the structural *NEAT1* RNA or phase separation proteins in paraspeckles will help develop new strategies for targeted therapies.

#### Chromatin Foci

In addition to phase separation related to paraspeckles involved in RNAs and proteins, phase separation formed at chromatin is common to regulate gene transcription and chromosome segregation. Phase separation formed at chromatin is usually affected by the surrounding microenvironment, such as nucleosome state and modification of histone. Exposing histone tails of nucleosome makes the interaction of inter-nucleosome tighter and thus promotes the phase separation formation by HP1 (Gibson et al., 2019; Sanulli et al., 2019). Phase separation prefers to form at low-density chromatin compared to high-density regions, referred to as heterochromatin. This preference caused by phase separation usually results in reorganization of chromatin and thus alters gene expression (Gibson et al., 2019). Many studies suggest that lncRNAs in specific chromatin loci also function as scaffolds to recruit chromatin-modifying complexes, promoting the epigenetic regulation of gene expression. X-chromosome inactivation (XIC) is a critical epigenetic mechanism for balancing gene dosage between XY and XX in eutherian mammals. Recent studies suggest that the process of X-chromosome inactivation is involved in phase separation mediated by *Xist* (Cerase et al., 2019). *Xist* drives phase separation by enriching chromatin remodeling factors, such as Spen, Ptbp1, HnrnpK, and PRC1/2 IDR-protein (Moindrot et al., 2015). This recruitment leads to deacetylation of histone and chromatin condensation. After inactivation, the X-chromosome is sequestered by specific interactions between *Xist* and Lamin-B receptor (Cerase et al., 2019). Pericentromeric heterochromatin (PCH) formation is also a phase separation process, mainly mediated by HP1α and lncRNA *MajSAT*. In these PCH foci, the R/G-rich domain of RNP protein SAFB is responsible for recognizing lncRNA *MajSAT*. SAFB-*MajSAT* interaction functions as a scaffold for the 3D organization of heterochromatin (Huo et al., 2020). What is impressive in this study is that, although the SAF family proteins SAFA/B have a similar functional domain, only SAFB confers the formation of PCH foci. The factor contributing to this difference is interesting for future studies. Telomeres, a special part of the chromosome, consist of DNA-protein complexes involved in chromosome end protection. It has been reported that many cancer cells can escape senescence by altering the length of telomeres, which is also termed alternative lengthening of telomeres (ALT). LncRNA TElomeric Repeat-containing RNA (LncRNA *TERRA*), transcribed at telomeres, is a main hallmark of ALT (Roake and Artandi, 2017; Bettin et al., 2019). Evidence has indicated that lncRNA *TERRA* acts as a scaffold to promote the recruitment of epigenetic modification factors (such as PRC2 and HP1) and diverse RBPs (such TLS/FUS and TRF2) (Deng et al., 2009; Takahama et al., 2013; Montero et al., 2018), which always appear in numerous phase separations. Simultaneously, lncRNA *TERRA* was reported to be enriched in ALT-associated PML body (APB), one of the promyelocytic leukemia (PML) bodies, which are nuclear membraneless organelles formed by LLPS and are involved in mitosis by recruiting multivalent proteins with small ubiquitin-like modification (SUMO) sites and SUMO-interacting motifs (SIMs) (Arora et al., 2014; Banani et al., 2016). A recent study reported an artificial model system where APB could form telomere cluster condensates by LLPS *in vivo*. During this process, BLM helicase and RAD52 are responsible for the formation of telomeres’ foci (Min et al., 2019). Considering the enrichment of lncRNA *TERRA* in APB and the interactions between lncRNA *TERRA* and epigenetic modification factors and RBPs, we speculate that lncRNA *TERRA* may also play a functional role in the telomere foci. However, the detailed mechanism needs to be further investigated. Of note, why both lncRNA *MajSAT* and LncRNA *TERRA*, repetitive RNAs, are preferred to be selected to participate in the formation of phase separation needs further exploration.

Corrective pairing and segregation of homologous chromosomes in meiosis are critical to producing haploids. LncRNA *sem2 RNA* helps Smp (sme2RNA-associated protein) protein form three chromosome loci and determine the specificity of chromosomal loci for fusion. It indicates the importance of Smp proteins in the accumulation of lncRNA and the critical role of lncRNA-mediated chromosome homologous pairing in *Schizosaccharomyces pombe* (Ding et al., 2019). In the fission yeast, the *meiRNA* plays a crucial role in recognizing and pairing homologous chromosomes during meiotic prophase. LncRNA *meiRNA* recruits Mmi1 protein to sem2 dot to promote meiosis, which is pivotal for selective elimination of meiosis-specific transcripts (Shichino et al., 2014). Enhancers and SEs are good partners to explain the bursting expression of genes. Recent studies reveal that enhancers, SEs, and eRNA may be involved in phase separation. Transcribed from bidirectional ncRNA, eRNAs act as enhancers and alter the chromosomal architecture during the transcription process (Li et al., 2013; Liu et al., 2014; Pnueli et al., 2015). Under the acute stimulation of 17β-estradiol (E2), eRNA and several TFs provide a conductive microenvironment for the assembly of enhancer RNA–dependent ribonucleoprotein (eRNP), regulating signal-inducible transcription (Nair et al., 2019). eRNAs wire DNA and TFs together and thus promote gene expression. RNA-exosome regulates the degradation and terminates transcription of eRNA lncRNA *CSR*, which coordinates SEs to promote the stability of chromatin in long range (Pefanis et al., 2015). eRNAs highly expressed in many cancers may be responsible for drug resistance by promoting related gene expression, which indicates that certain eRNAs can be diagnostic markers and targets for cancer treatment (Zhang et al., 2019).

The diverse functions of lncRNA combined with phase separation in chromatin loci show a spectacular panoramic view for understanding the regulation of gene expression at the transcriptional level. The inactivation and segregation of chromatin and gene bursting expression can be well-interpreted by the phase separation model. The chromatin loci formed by phase separation through specific proteins and lncRNA provide us with new strategies to explore the abnormal cellular processes and develop novel therapy.

#### Nuclear Stress Bodies

The nucleoplasm is a natural pool for diverse nuclear bodies to regulate gene expression (Chujo and Hirose, 2017; Ninomiya and Hirose, 2020). Nuclear bodies accumulated at specific nucleus sites affect the biogenesis, maturation, storage, and sequestration of specific proteins and RNAs, thus altering cellular events to respond to stress stimuli. Under thermal stress, lncRNA *HSATIII* acts as the structural scaffold for the HNRNPM and SAFB foci formation and retains numerical RBPs to regulate gene expression (Aly et al., 2019). To respond to DNA double-strand breaks (DSB), damage-induced long non-coding RNA (*dilncRNA*) is synthesized at DSB foci, also called DNA-damage-response (DDR) foci. *DilncRNA*, together with DDR proteins, such as 53BP1, promotes the formation of DDR foci to regulate the transcriptional activity of genes mediating the DSB signal pathway (Pessina et al., 2019). In a wide range of cancers, lncRNAs and related RBPs are often aberrantly transcribed. LncRNA *PNCTR* recruits RBP PTBP1 to form a nuclear body called peri-nucleolar compartment (PNC), where lncRNA *PNCTR* modulates cellular localization of PTBP1 by changing the splicing of PTBP1, an activator of the intrinsic branch of apoptosis. The alteration of PTPB1 cellular localization results in its inhibition and thus promotes cell survival (Yap et al., 2018). In colon cancer, upregulated lncRNA *TNBL* is accumulated at the subset of NBL2 loci and forms dense aggregates, which sequesters SAM68 RBPs and nucleic acids. This SAM68 nuclear body may disrupt nuclear organization (Dumbovic et al., 2018). LncRNAs involved in many events in nuclear bodies can enrich our insights to better understand the function of lncRNAs in phase separation.

## Prospective

This review mainly summarized the current findings on phase separation and the potential roles of phase-separation related lncRNAs. The formation of phase separation involves multiple molecules, such as RNAs, proteins, and related chromatin. Maintenance of phase separation relies on its surrounding environments, such as pH, temperature, and the concentration of salt solution. Sometimes phase transition is largely determined by the sequence of RNAs, proteins, and the PTM of proteins. Phase separation has expanded our understanding of biochemical reactions and biological processes in membraneless organelles. LncRNAs mainly function as architectural scaffolds for diverse RNA and protein interaction in this process. Phase separation coordinating lncRNAs in multiple nuclear bodies are mainly involved in regulating gene expression, chromatin remodeling, RNA splicing, and homologous chromosome separation in the nucleus. However, lncRNAs involved in cytosolic phase separation are less reported. Several studies have revealed that phase-separation related lncRNAs in cytoplasm participate in signaling transduction (Lee et al., 2016; Tichon et al., 2016). This evidence inspires us to explore more about cytosolic lncRNAs-mediated phase separation. Combining the function of lncRNAs and phase separation together, current studies on both are only the tip of the iceberg. Major questions have yet to be answered in these emerging fields about phase separation and lncRNAs. The most concerning problem is identification of the factors that confer the special components in phase droplets. Proteins contained with LDR, PrLD, or RNA with a repetitive sequence are more likely to form phase separation. Maybe the distribution of net charge and the advanced structure of RNAs and proteins are major factors, which have a great influence on multivalent interactions. Other environmental factors, such as pH, temperature, and the concentration of salt solution, are also important for phase separation formation. The second problem is how the subcellular localization of lncRNAs and phase separation-related proteins affects the phase separation formation. It seems that more functional phase separated droplets tend to form in the nucleus, which is mostly related to the formation of heterochromatin. What factors contribute to this preference needs to be further elucidated. Numerous nuclear bodies exert different roles in gene expression and epigenetic regulation. Why different lncRNA are selected in different functional phase separation droplets needs to be further explored. Moreover, which factors and signaling pathways are involved in the dynamically assembled and disassembled phase separation droplets upon different environmental stress is of significance. It is also of great importance to precisely identify the role of lncRNAs in sensing stress stimulations, signal transduction, and maintenance of phase separation. Such discoveries will help better understand and develop better therapeutic treatments for phase-separation related diseases.

## Author Contributions

AL contributed to the study design and data analysis. JLuo wrote the manuscript. LQ and FG contributed to the figure and table design. AL, JLiu, and JLin edited the manuscript. All authors contributed to the article and approved the submitted version.

## Conflict of Interest

The authors declare that the research was conducted in the absence of any commercial or financial relationships that could be construed as a potential conflict of interest.
